# Non-surgical treatment of anterior cruciate ligament tears with percutaneous bone marrow concentrate and platelet products versus exercise therapy: a randomized-controlled, crossover trial with 2-year follow-up

**DOI:** 10.1186/s12891-025-09153-2

**Published:** 2025-09-30

**Authors:** Christopher J. Centeno, Dustin R. Berger, John Pitts, Jason Markle, Andrew J. Pelle, Matthew Murphy, Ehren Dodson

**Affiliations:** 1 Centeno-Schultz Clinic, Broomfield, CO 80021 USA; 2 Regenexx, LLC, Research and Development, Broomfield, CO 80021 USA

**Keywords:** Anterior cruciate ligament, ACL tear, Bone marrow concentrate (BMC), Platelet-rich plasma (PRP), Mesenchymal stem cells; randomized trial; regenerative medicine, Exercise therapy

## Abstract

**Background:**

Anterior cruciate ligament **(**ACL) reconstruction (ACLR) surgery remains the prevailing standard of care for complete ACL ruptures but is not without risk. The aim of this study was to compare non-surgical, autologous bone marrow concentrate (BMC) with platelet products compared to exercise therapy for partial and complete, non-retracted ACL tears without meniscus injuries.

**Methods:**

In this randomized-controlled, crossover trial, patients received either exercise therapy or an injection of BMC with platelet products to treat ACL tears. After 3 months, if patients randomized to the exercise group were not satisfied with their improvement, they could crossover to receive the BMC treatment. Patients reported outcomes were completed at pre-treatment, 1-, 3, 6-, 12-, and 24-months of the Numeric Pain Scale (NPS), the International Knee Documentation Committee (IKDC) subjective knee form, Lower Extremity Function Scale (LEFS), and a modified Single Assessment Numeric Evaluation (SANE). MRIs at pre- and post-treatment were assessed using ImageJ software analysis. Complications and surgeries were assessed at each time point.

**Results:**

Patients in the BMC group reported significantly greater change scores (from pre-treatment) on LEFS (*P* = 0.027), and SANE (*P* = 0.007) at 3 months compared to the exercise group. No significant differences were found between baseline and/or 1- month and 3-month follow-ups for any outcome measure (*P* > 0.05) in the exercise therapy group. When followed through 2 years, patients who received BMC treatment demonstrated sustained improvements in function and decreases in pain, with patients reporting a median of subjective improvement (SANE) of 90% at the final follow-up. Significant negative correlations were found between age of injury and ΔIKDC ΔLEFS, and SANE (*P* < 0.05) at the 24-month follow-up with lower ΔPROMs reported by those with older tears the age of tear (> 12 months). Post-treatment MRI provided evidence of significant improvements in ACL integrity. No serious adverse events were reported. Four patients did not respond to BMC treatment and underwent ACL reconstruction without any new reports of knee injuries.

**Conclusions:**

The significant improvements in function, pain, and overall improvement in patients receiving BMC and platelets provide evidence of a viable alternative treatment option for patients with ACL tears.

**Trial registration:**

NCT01850758; A Randomized Controlled Trial of Regenexx-SD Versus Exercise therapy for Treatment of Partial and Complete, Non-retracted Anterior Cruciate Ligament Tears. Registered May 7, 2013.

**Supplementary Information:**

The online version contains supplementary material available at 10.1186/s12891-025-09153-2.

## Introduction

The anterior cruciate ligament (ACL) serves as a crucial stabilizer of the knee joint, preventing anterior translation of the tibia and restraining tibial rotation [1]. ACL injuries are common, with over four hundred thousand ACL reconstruction (ACLR) surgeries performed each year in the United States alone, remaining the prevailing standard of care for complete ACL ruptures [2]. However surgical approaches are not without limitations and associated risks, including the possibility of graft failure, failure-tear, persistent instability, cartilage injury and arthrofibrosis [3]. Moreover, a primary concern following ACL injury, regardless of the treatment approach, is the development of knee osteoarthritis (OA), as extensive research has established a markedly elevated risk of OA in knees with ACL injuries, when compared to uninjured contralateral knees, underscoring long-term consequences on joint health [4].

Return to sport is a primary driving factor for many patients seeking to undergo ACLR surgeries. However, one-year return to sports rates vary considerably, ranging from 33 to 92% [5], with a systematic review of the literature reporting a post-surgical return to sports rate of 81%, a return to preinjury level of 65%, and a return to competitive sport level of 55% [6]. Given the complex nature of surgical ACLR and associated risks, the apparent lack of prevention of progressive knee OA, and variable return to sports, there exists a compelling need to explore less invasive and alternative treatment options for ACL injuries.

In recent years, the field of orthobiologics has witnessed significant progress, particularly in the use of platelet-rich plasma (PRP) and bone marrow concentrate (BMC) for the treatment of various musculoskeletal conditions [7–9]. Although exact mechanisms of action are still being elucidated, the cells and platelets concentrated within PRP and BMC release cytokines, exosomes, and growth factors that are believed to initiate cellular signaling to help mobilize a healing cascade [10, 11]. Additionally, BMC contains a population of connective tissue progenitor cells, commonly referred to as mesenchymal stem cells (MSCs), which are thought to utilize paracrine mediated effects to provide benefit in skeletal tissue healing and repair [7, 8, 12]. The promise of healing native ACLs lies in the potential to preserve the natural orientation, knee kinematics, and proprioceptive properties. Encouraging results from animal models incorporating bone marrow MSCs into scaffolds or matrices for ACL injury repair have fueled interest in its clinical application [13], though human studies remain relatively limited. There have been several randomized controlled trials of BMC used in combination with surgical graft reconstruction, one showing improved MRI ACL signal at three months and improved function at nine months [14] and the other showing decreased laxity at 6 months [15]. Wang et al. provided evidence that an injection of allogeneic human mesenchymal precursor cells following ACLR improved symptoms and halted joint degeneration, helping prevent secondary OA [16]. Two case series utilizing BMC combined with PRP via percutaneous injection of the ACL tear without surgical reconstruction have reported increased ACL integrity and functional improvements [17, 18].

The primary aim of this study is to compare the improvement in self-reported outcome measures between patients receiving percutaneous BMC treatment composed of a combination of autologous BMC and platelet products and those patients performing exercise therapy for the treatment of partial thickness and full thickness, non-retracted ACL tears. A midterm analysis of this patient population revealed promising results for patients treated with BMC related to reported function and ACL integrity [19]. Findings from this completed clinical trial are reported here.

## Methods

### Study design

This was a randomized-controlled, open-label, crossover design trial. Study participants were recruited from the local community by way of orthopedic pain practice and through radio, print, website, and social media advertisements from August 2013 to February 2020. Ethics approval for the study protocol was obtained from the International Cellular Medicine Society (OHRP Registration #IRB00002637). Study was registered on ClinicalTrials.gov (NCT01850758) on May 7, 2013. Patients that met the study criteria were consented and enrolled into the trial and randomized to either receive treatment with a combination of autologous BMC, PRP, and platelet lysate (PL) (BMC treatment group) or undergo an exercise regimen (exercise therapy group). Inclusion criteria comprised being aged 18–65, and having: knee pain, swelling, and/or functional disability; failed conservative therapy for at least three months; a physical exam consistent with laxity in the ACL (Positive Lachman’s test); a positive diagnostic MRI with at least one-third of the ACL at any point along its length with high signal (minimum of Grade 2 tear); and full range of motion. Exclusion criteria included having: a massive retracted ACL tear; previous surgery to the ACL; received any injection therapies to the knee within the previous three months; concomitant meniscal tearing and/or cartilage injury considered the pain generator; concomitant ligamentous (PCL or LCL) tears; symptomatic lumbar spine pathology; Kallgren-Lawrence grade II or greater knee OA; inflammatory or autoimmune based joint disease or lower extremity pathology; previously tested positive or treated for malignancy; quinolone or statin induced myopathy/tendinopathy; severe neurogenic inflammation of the cutaneous nerves about the knee or thigh; contraindications for MRI; a condition representing a worker’s compensation case or currently involved in health-related litigation; is pregnant; bleeding disorders; is currently taking anticoagulant or immunosuppressive medication; chronic use of opioids; a documented history of drug abuse within six months of treatment; an allergy to study medications; and any other condition that in the opinion of the investigator would preclude patient enrollment.

All patients signed written informed consent. The study protocol was originally designed to have equal group allocation, with twenty-five patients randomized to each group, for a total of fifty. This sample size was adequate to test for an 11.5-point IKDC difference between treatment groups in mean change from baseline to the 3-month follow-up with approximately 70% power (α = 0.05). Treatment group randomization occurred using a computer-generated randomization program, and patient allocation to each group was revealed by opening sequentially numbered envelopes containing study group based on order of enrollment. About halfway through the enrollment period, after preliminary analysis showed a larger than anticipated effect size, the study protocol was amended to include a 2:1 group allocation for improvement of patient recruitment, resulting in thirty-four patients randomized to the BMC treatment group and seventeen randomized to the exercise therapy group.

### Exercise therapy protocol

Patients randomized to the exercise therapy group met with a physical therapist and were given two sets of knee strengthening and stability exercises to perform in the form of take-home instructional handouts; one for the first six weeks, and a separate one for the subsequent six-week period (Supplementary 1). Protocol compliance was confirmed with research staff after approximately four to six weeks into the exercise therapy period. If, after three months of exercise therapy, patients demonstrated a lack of improvement in pain or function, they were provided the opportunity to cross over to receive BMC treatment and were followed for an additional twenty-four months.

### Preparation of BMC and platelet products

Patients randomized to the treatment group had bone marrow harvested from their posterior superior iliac crest, under fluoroscopic or ultrasonic guidance, into heparinized syringes (1,000 IU per mL bone marrow). A total of 6 to 8 sites (3–4 sites per side) were used to harvest 60–90 mL of bone marrow aspirate (BMA), obtaining approximately 5–15 mL per site to maximize the recovery of progenitor cells [20]. The resultant BMA was manually processed into 2 to 5 mL of bone marrow concentrate (BMC) by trained laboratory personnel using multi-step centrifugation (200 g for 6 min) and subsequently isolating the compact central layer of nucleated cells within a biosafety cabinet (ISO-5) and surrounding cleanroom environment (ISO-7). More specifics on these preparations have been published [19]. A small representative sample (< 0.1 mL) of BMC was retained for cellular analysis using an automated cell counter (TC20, BioRad) following removal of red blood cells by osmotic lysis. In addition, approximately 60 mL of anticoagulated whole blood was obtained by venipuncture to manually prepare PRP and PL. Blood was centrifuged (200 g for 10 min) to isolate leukocyte-poor plasma, which was subsequently centrifuged (2,300 g for 6 min) to collect platelets for resuspension in a reduced plasma volume (3 to 4 mL or a 15 to 20-fold reduction in volume). A portion of the PRP was frozen at −80 °C and thawed to prepare PL [21].

### BMC treatment protocol

**Fig. 1:**
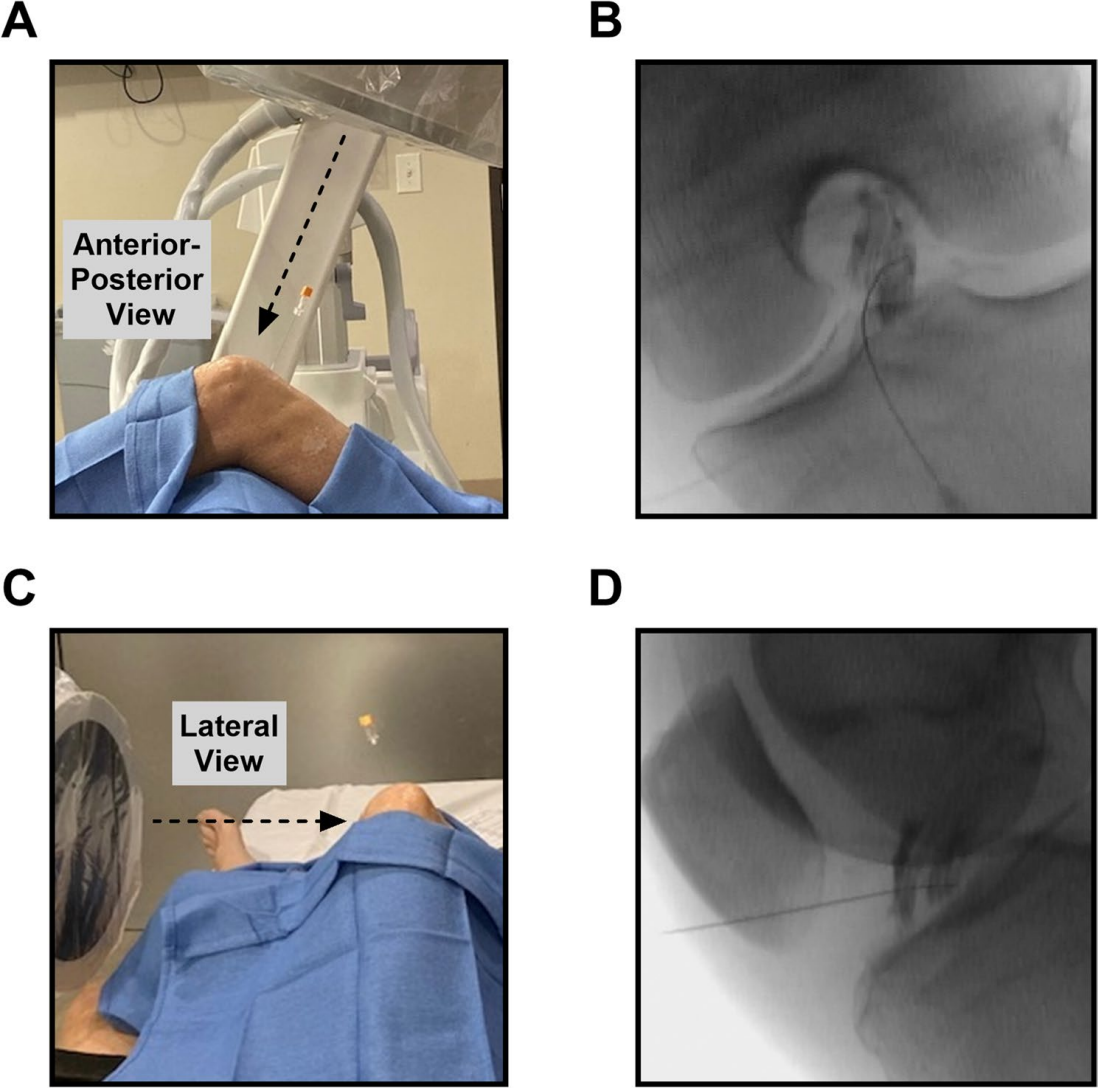
Injection approach confirmed with contrast flow pattern of both anteromedial (AM) and posterolateral (PL) bundles of the ACL. (**A**) (**B**) Anterior-Posterior view (**C**) (**D**) Lateral view

Extensive details of the BMC injection protocol have been reported [19]. After a BMA was performed and BMC was created in the lab, the patient was placed in a supine position with the knee being treated at approximately 45 degrees of flexion. Under fluoroscopy, in biplanar view, contrast (Iodexidol, NDC#0407-2223-06) flow was used to confirm appropriate intra-ligament needle placement, ensuring that the contrast flow pattern of both anteromedial (AM) and posterolateral (PL) bundles of the ACL traveled between the radiographic insertion and origin landmarks. If not achieved, the needle was redirected using guidance and the injection process repeated until full coverage was noted. Once placement was confirmed, 2 to 3 mL of an equal mixture of BMC, PRP, and PL was injected directly into the ligament. (Fig. 1). The needle was subsequently withdrawn from the ligament and while still in the joint, approximately 2 to 4 mL of an equal mixture of PRP and PL along with any remaining BMC was injected into the intra-articular space. No bracing was required. All patients were encouraged to follow the following rehab protocol and participate in activities as tolerated. Patients were directed to refrain from activities that caused more than 2 out of 10 in pain (on a 10-point scale) for the duration of rehabilitation. For the first month, patients were directed to perform range of motion exercises, light strength training, and balance training while protecting the knee. Afterwards (weeks 5–12), patients participated in resistance training, targeting the hip abductor and hamstring with light squats and leg presses, along with core strengthening using a balance board. If not experiencing pain, straight jogging, single leg exercises, and progression to combo strength/balance exercises were encouraged. Over the next eight weeks (weeks 13–20), patients began guided sport-specific movements and noncutting sports. Finally (weeks 21–52), the Santa Monica Sports Medicine Prevent Injury and Enhance Performance program was recommended and ultimately returned to full sport only with physician clearance.

### Clinical outcome measures

Patient-reported outcome measures (PROMs), including the International Knee Documentation Committee subjective knee form (IKDC), the Lower Extremity Function Scale (LEFS), the Numeric Pain Scale (NPS), a modified version of the Single Assessment Numeric Evaluation score (SANE), and any occurrence of adverse events or surgeries were completed at baseline and follow-ups of 1-, 3-, 6-, 12- and 24-months. The IKDC contains questions related to the extent of one’s ability to perform various physical sports activities without problematic knee symptoms, while the LEFS assesses one’s perceived difficulties with everyday activities [22, 23]. The NPS is an eleven-point scale that asks patients to rate their recent pain, where zero is reflective of no pain and ten represents the worst possible pain [24]. The modified SANE states, “Compared to your condition prior to the procedure, what percent difference have you seen in your condition, from − 100% worse to 100% better; 0% indicates no change?” and was only collected at the 1- through 24-month follow-up visits [21].

Return to sport was measured indirectly by utilizing patient responses to specific questions on the IKDC subjective knee form, including “What is the highest level of activity you can perform without significant giving way in your knee?” (IKDC question 7), and “What is the highest level of activity you can participate in on a regular basis?” (IKDC question 8). Answers for these questions range from “very strenuous activities like jumping or pivoting as in basketball or soccer” and “strenuous activities like heavy physical work, skiing or tennis”, to “moderate activities like moderate physical work, running or jogging”, “light activities like walking, housework or yardwork”, and “unable to perform any of the above activities due to giving way of the knee”.

### ACL tear classifications

Pre-treatment MRIs were used by two blinded reviewers to classify baseline ACL tears on the sagittal plane for all patients. Based on clinical experience with treating ACL tears using this method, it was determined that the traditional binary tear classifications were insufficient to guide treatment decisions, hence a more detailed classification system was validated [19]. This includes a partial tear (PT), consisting of a grade II ligamentous tear with high MRI signal in less than half the ACL width, a complete non-retracted type 2 tear (CNR2) consisting of a grade III ligamentous tear with high MRI signal in more than half the ACL width, but less than five millimeters longitudinally and having otherwise normal fiber morphology (diffuse signal on MRI and thicker ACL appearance), a complete non-retracted type 1 tear (CNR1) similar to a CNR2 tear, but presenting with ‘blown out’ fiber morphology, and a complete retracted tear (CR) consisting of a grade III ligamentous tear with a complete absence of fibers connecting the ends of the ligament and retraction of those fibers of greater than five millimeters [19].

### Image analysis

Patients underwent magnetic resonance imaging (MRI) for initial evaluation to confirm meeting study inclusion criteria, and most received a post-BMC treatment MRI of their knee at approximately the 6-month follow-up, with similar instrument and imaging protocol settings specified. The timeframe for post-treatment imaging was extended in July 2017, due to the original 3-month duration being too early to optimally capture ACL changes on MRI. Cross sectional, sagittal or axial MRI images best presenting the ACL were identified (RadiAnt DICOM Viewer, Medixant, Poland) from pre- and post-BMC treatment visits and utilized for a previously recognized imaging analysis comparing pixel intensities as a representative for ACL integrity [17, 19]. Linear histogram stretching was used for image normalization, and a region-of-interest (ROI) was positioned around the ACLs by a single blinded observer (JM) using imaging software (Fiji/ImageJ 1.54f, National Institutes of Health, USA) [25]. Mean grey pixel intensities from each region-of-interest were measured for pairwise comparison, with lower signal intensities indicative of a more intact ACL ligament.

### Statistical analysis

Descriptive statistics were provided as medians with interquartile range or averages with standard deviations depending on normality of data assessed using the D’Agostino and Pearson test. Changes in PROMs (ΔPROMs) were calculated by subtracting baseline from follow-up measures. ΔPROMs were compared at the 3-month follow-up between patients randomized to the exercise group and those receiving BMC treatment using Mann-Whitney tests. Wilcoxon matched-pairs signed rank tests, corrected for multiple comparisons using the Holm-Sidak method, were used to measure patient improvement for three months following exercise and twenty-four months following BMC treatment. Spearman correlations were used to measure associations between ΔPROMs at the 24-month follow-up and the age of ACL injury. Pre- and post-BMC treatment MRI mean grey pixel intensities within ROIs outlining ACLs were compared using a paired t-test. Patient outcomes based on tear grade classification groups were compared using a Kruskal-Wallis test. All results were considered significant at *P* < 0.05 (Prism 10.2.0, GraphPad Software, USA).

## Results

**Fig. 2:**
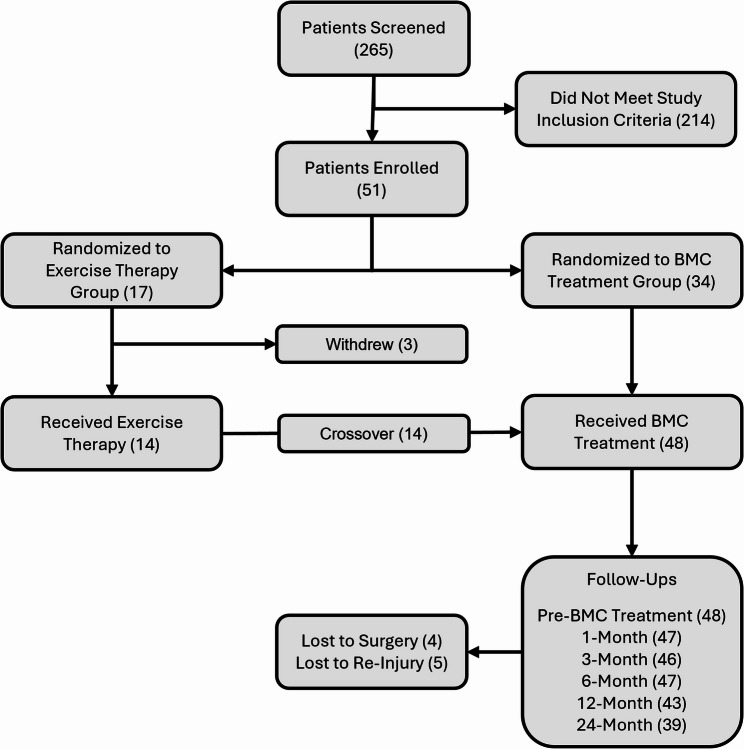
Study flow diagram, *n* = displayed

Two hundred sixty-five patients were screened for study eligibility, with fifty-one meeting inclusion criteria and enrolling. Thirty-four patients were randomized to receive autologous BMC treatment and seventeen to complete exercise therapy. Three patients in the exercise therapy group withdrew from the study prior to starting therapy, and the remaining fourteen patients underwent at least three months of exercise. All of these patients crossed over to receive BMC treatment (Fig. 2). Demographics were similar for both patient groups, though more males than females were randomized to receive BMC treatment (Table 1). Five patients were withdrawn owing to re-injuries of their knee within the two-year follow-up period (two ACL retears, one meniscus injury, one LCL injury, one motor vehicle accident), and another four patients went on to undergo ACLR and were considered treatment failures (Table 2). All patients that sustained a new injury/re-tear except the 3-month meniscus tear had shown evidence of healing prior to new injury. Further, three patients received repeat treatment of autologous BMC after having an incomplete or non-response to initial treatment (at 6, 9 and 12 months) and twenty-one patients received an autologous PRP booster (at a median of 7 months) after experiencing a plateau in their healing and persistent symptoms in their ACL pathology. Two of the four patients that underwent ACLR during the study had received additional treatment of BMC or PRP, while the other two declined additional treatment. Patients’ ACL tears were classified and listed in Table 3. Adverse events (AEs) were monitored at all follow-ups, without any report of a serious adverse event. Several AEs reported included one case of knee effusion after the injection that required aspiration, a case of temporary paresthesia corresponding to knee brace use after treatment, and one case of residual pain at the BMA site that resolved on its own.

**Table 1:** Demographics of study participants

Characteristic	Exercise Therapy	BMC Treatment	*P* Value
Gender	7 Females 7 Males	9 Females 25 Males	0.178^‖^
Age (years)	40.9 ± 9.5	36.8 ± 10.6	0.211^†^
BMI (kg/m^2^)	24.8 ± 4.0	24.2 ± 2.7	0.549^†^
Height (inches)	67.6 ± 4.8	69.0 ± 3.3	0.256^†^
Weight (lbs)	161.5 ± 29.7	164.4 ± 24.2	0.722^†^
Baseline IKDC	51.2 ± 16.5	58.7 ± 17.9	0.198^†^
Baseline LEFS	54.1 ± 11.5	56.4 ± 13.3	0.588^†^
Baseline NPS	2.0 [1.0–4.0]	2.0 [1.0–3.5]	0.671^‡^
AOI (months)	6.0 [5.8–8.0]	5.0 [4.0–16.0]	0.742^‡^
Total Cells (10^6^)	659 [521–1050]*	643 [475–1100]	0.827^‡^

Values presented as mean ± standard deviation or median [interquartile range]. *IKDC* Internation Knee Documentation Committee subjective knee form, *LEFS* Lower Extremity Functional Scale, *NPS* Numeric Pain Scale, *BMI *Body Mass Index, *AOI *Age of Injury. Fisher’s exact test^‖^, Unpaired t-test^†^, Mann-Whitney test^‡^. Cells counted upon crossing over to BMC treatment*

**Table 2:** Study participants lost to injury (two ACL re-tears) and ACL reconstruction throughout the two-year follow-up period

Follow-Up	Injury	Surgery
1-Month	-	-
3-Month	1	-
6-Month	-	-
12-Month	1	2
24-Month	3	2
Total	5	4

**Table 3:** Prevalence of ACL tear types

ACL Tear Classification	(*n*)	(%)
Partial	1	2.1%
Complete Non-Retracted Type 1	27	56.3%
Complete Non-Retracted Type 2	16	33.3%
Complete Retracted	4	8.3%

**Fig. 3:**
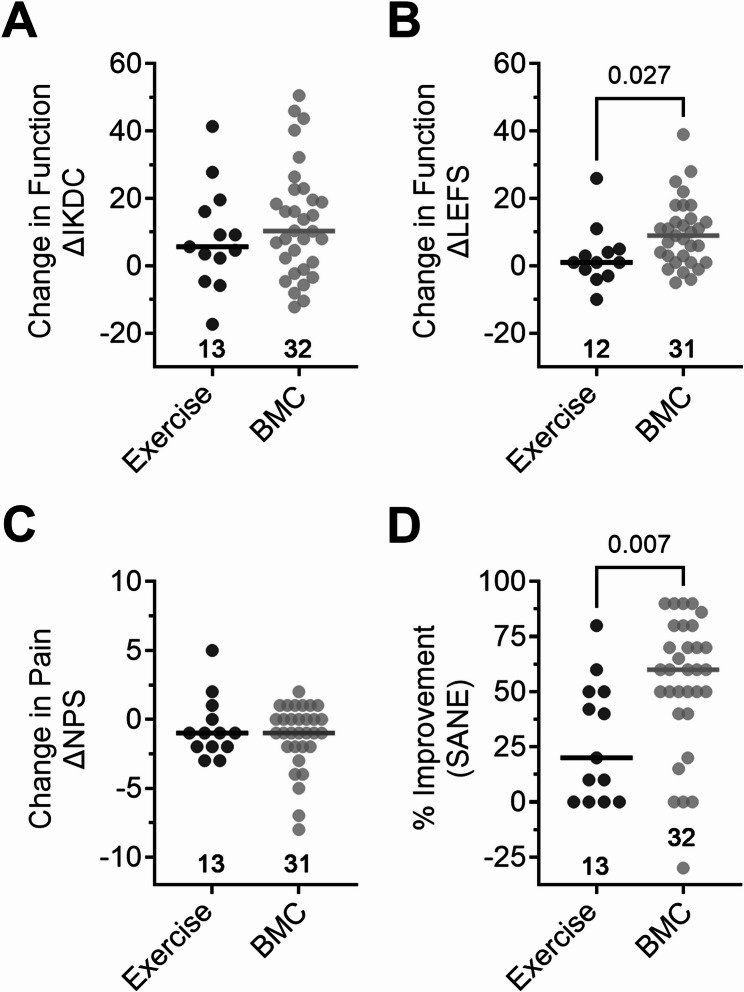
ΔPROMs for IKDC (**A**), LEFS (**B**), NPS (**C**), and SANE (**D**) from the exercise therapy group compared to the BMC treatment group at the 3-month follow-up. ΔPROMs were compared between patient groups using Mann-Whitney tests; lines represent median values, *n* = displayed

At the 3-month follow-up, patients treated with autologous BMC reported significantly greater changes in several PROMs than reported by those in the exercise therapy group (Fig. 3). LEFS increased a median of 9 points [IQR 1 to 14] in the BMC group compared to a median of 1 point [IQR − 2.5 to 4.75] in the exercise group (*P* = 0.027). Similarly, the median SANE was 60% [IQR 42.5–77.5%] in the BMC group and 20% [IQR 0–50%] in the exercise group (*P* = 0.007). Yet, other ΔPROMs were not significantly different in both groups, as IKDC increased a median of 10.4 points [IQR − 0.6 to 21.9] and 5.7 points [IQR − 1.2 to 17.9] and NPS decreased a median of 1 point [IQR − 2.0 to 0.0] and 1 point [IQR − 2.0 to 0.5] in the BMC group and exercise group, respectively (*P* > 0.05).

**Fig. 4:**
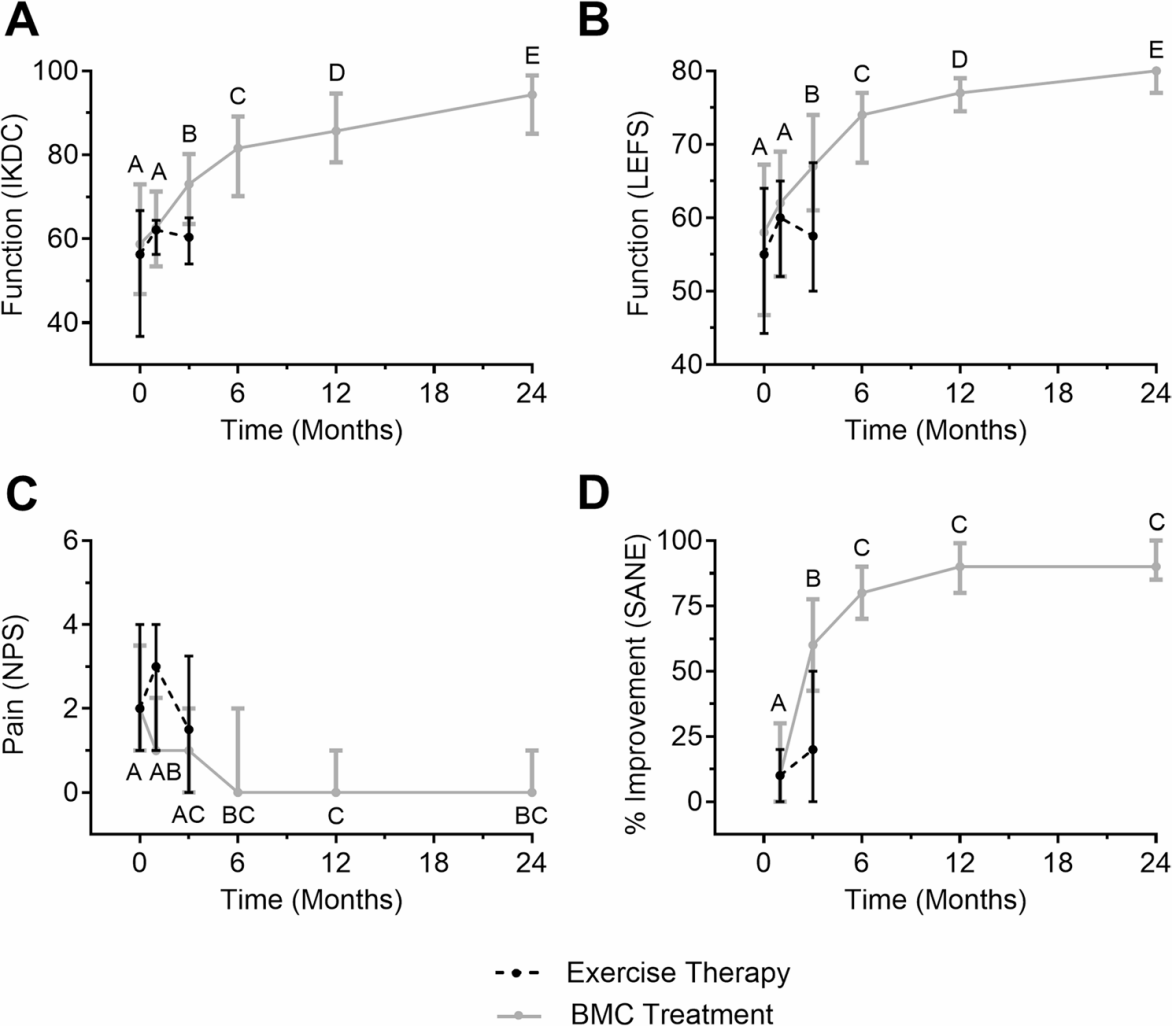
Exercise therapy group and BMC treatment group PROMs over the duration of the study period. Median values and interquartile ranges for IKDC (**A**), LEFS (**B**), NPS (**C**), and SANE (**D**) are shown. Within group PROMs were compared over time using multiple Wilcoxon matched-pairs signed rank tests with Holm-Sidak correction. BMC treatment group follow-ups sharing a letter are statistically indistinguishable, whereas those significantly different are noted with different letters (*P* < 0.05)

No significant pairwise differences (adjusted *P* > 0.05) were found between baseline, 1-, or 3-month for all PROMs from the exercise therapy group (Table 4). In contrast, BMC group IKDC, LEFS, and SANE values at the 3-month follow-up were significantly greater than those at baseline (Table 5). Moreover, while significant improvement in NPS and SANE leveled off after six months, IKDC and LEFS values continued to increase significantly throughout the duration of the study period (Fig. 4). Patients originally randomized to the exercise therapy group also reported significant improvements in PROMs upon crossing over to BMC treatment, up to the 24-month follow-up (Supplementary 1 and 2). Those receiving a booster injection reported significantly lower SANE values at the 12- and 24-month (*P* < 0.05) follow-ups than patients without, and changes in IKDC and LEFS trended lower in this patient population (*P* > 0.05) (Supplementary 3). Further, those receiving booster injections had a longer age of injury (AOI), although non-significant (*P* > 0.05); the group without booster injections had median AOI of 6 months versus an AOI of 8.5 months for those that received a booster injection.

**Table 4:** Exercise therapy group proms

Exercise Therapy			
PROM	Baseline	1-Month	3-Month
IKDC	56.3 [36.8–66.7] *n* = 13	62.1 [56.3–64.4] *n* = 11	60.4 [54.0–65.0] *n* = 14
LEFS	55.0 [44.3–64.0] *n* = 12	60.0 [52.0–65.0] *n* = 11	57.5 [50.0–67.5] *n* = 14
NPS	2.0 [1.0–4.0] *n* = 13	3.0 [1.0–4.0] *n* = 11	1.5 [0.0–3.3] *n* = 14
SANE	n/a	10.0 [0.0–20.0] *n* = 11	20.0 [0.0–50.0] *n* = 13

Values presented as median, [interquartile range], n = displayed. No pairwise PROM differences between follow-ups via Wilcoxon matched-pairs signed rank test with Holm-Sidak correction (P > 0.05).

**Table 5:** BMC treatment group proms

BMC Treatment						
PROM	Baseline	1-Month	3-Month	6-Month	12-Month	24-Month
IKDC	58.8 46.8 to 73.0]^A^ *n* = 34	62.7 [53.4 to 71.3]^A^ *n* = 34	73.0 [63.5 to 80.2]^B^ *n* = 32	81.6 [70.2 to 89.1]^C^ *n* = 33	85.7 [78.2 to 94.6]^D^ *n* = 30	94.3 [85.1 to 98.9]^E^ *n* = 25
LEFS	58.0 [46.8 to 67.3]^A^ *n* = 34	62.0 [52.0 to 69.0]^A^ *n* = 33	67.0 [61.0 to 74.0]^B^ *n* = 31	74.0 [67.5 to 77.0]^C^ *n* = 33	77.0 [74.5 to 79.0]^D^ *n* = 30	80.0 [77.0 to 80.0]^E^ *n* = 24
NPS	2.0 [1.0 to 3.5]^A^ *n* = 33	1.0 [1.0 to 2.3]^AB^ *n* = 34	1.0 [0.0 to 2.0]^AC^ *n* = 32	0.0 [0.0 to 2.0]^BC^ *n* = 33	0.0 [0.0 to 1.0]^C^ *n* = 30	0.0 [0.0 to 1.0]^BC^ *n* = 25
SANE	n/a	10.0 [0.0 to 30.0]^A^ *n* = 33	60.0 [42.5 to 77.5]^B^ *n* = 32	80.0 [70.0 to 90.0]^C^ *n* = 33	90.0 [80.0 to 99.0]^C^ *n* = 30	90.0 [85.0 to 100.0]^C^ *n* = 25

Values presented as median, [interquartile range], n = displayed. PROM-specific follow-up timepoints sharing a letter are indistinguishable via Wilcoxon matched-pairs signed rank test with Holm-Sidak correction (P > 0.05), whereas values with different letters were significantly different (P < 0.05).

**Fig. 5:**
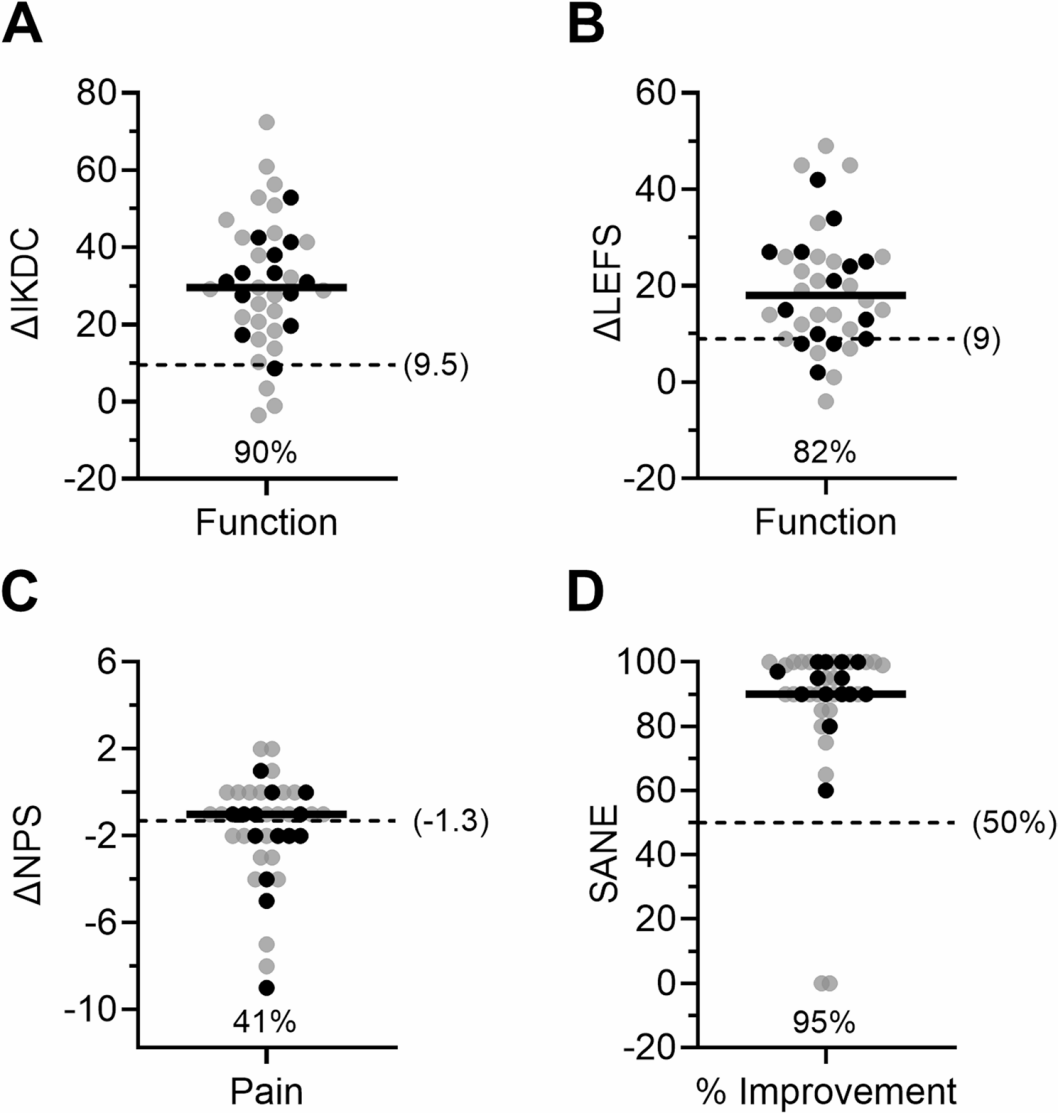
ΔPROMs at the 24-month follow-up for IKDC (**A**), LEFS (**B**), NPS (**C**), and SANE (**D**) compared to MCIDs reported in the literature. Percentages indicate the portion of patients reporting ΔPROM values equal to or greater than the MCID (less than the MCID for NPS, since decrease indicates improvement). Black dots represent patients from the exercise therapy group after crossing over to BMC treatment. Lines represent median values, *n* = 39 (*n* = 38 for ΔLEFS)

Most patients reported ΔPROMs for IKDC, LEFS, and SANE that met or exceeded published MCIDs at the 24-month follow-up. Of all patients receiving BMC treatment (BMC group + exercise crossover group) and providing 24-month outcomes, 90% reported ΔPROMs greater than the IKDC MCID of 9.5 and 82% reported ΔPROMs equal to or greater than the LEFS MCID of 9 [26, 27]. Though only 44% of patients met or exceeded the NPS MCID of −1.3, 49% reported baseline pain levels of zero or one and thus could not meet the NPS MCID [28]. Approximately 95% of patients met a 50% threshold in modified SANE (Fig. 5). Patient outcomes at 24 months did not differ significantly when comparing tear grade classification groups (*P* > 0.05). In addition, many patients reported a return to sport two years after BMC treatment, as measured from the top two responses (‘very strenuous activities’ and ‘strenuous activities’) to questions #7 and 8 on the IKDC subjective knee form. Very strenuous activities, including jumping or pivoting, as in basketball or soccer, was reported by 59% (23 of 39) of patients and strenuous activities, including heavy physical work, skiing, or tennis were reported by 28% (11 of 39) of patients as the highest activity able to be performed without significant giving way in the knee (IKDC question 7), representing a total of 87% of the patients. Similarly, 41% (16 of 39) and 38% (15 of 39) of patients responded with very strenuous activities, and strenuous activities, respectively, as the highest level of activity participated in on a regular basis (IKDC question 8), representing a total of 80% of patients responding at the 24-month time point.

**Fig. 6:**
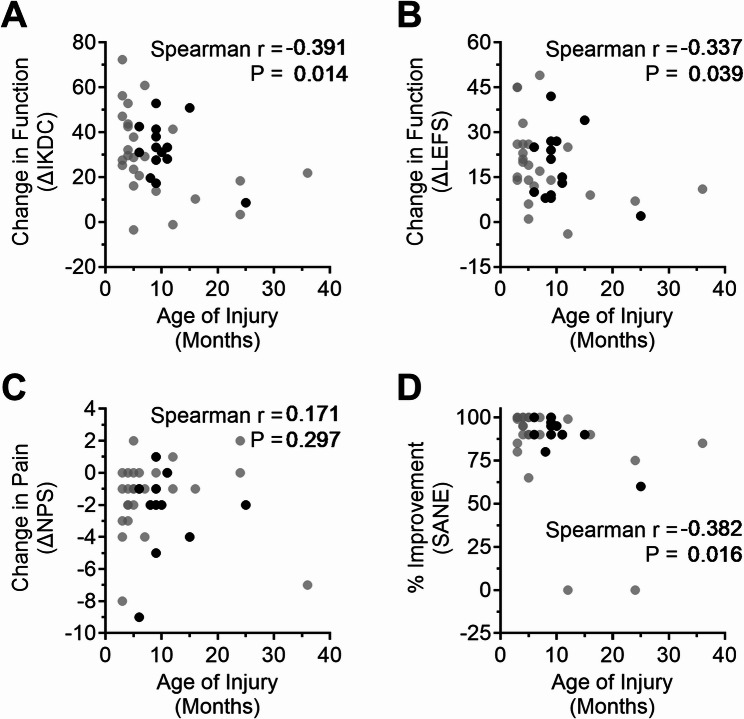
Associations between ΔPROMs at the 24-month follow-up for IKDC (**A**), LEFS (**B**), NPS (**C**), and SANE (**D**) and the age of ACL injury. Black dots represent patients from the exercise therapy group after crossing over to BMC treatment. Spearman correlation, *n* = 39 (*n* = 38 for ΔLEFS)

Three of four patients reporting reconstructive surgeries within the 24-month follow-up period had torn their ACL more than one year prior to receiving BMC treatment, prompting an inquiry into possible association(s) between ΔPROMs and age of injury. Significant negative correlations were found between age of injury and ΔIKDC (*r* = −0.391, *P* = 0.014), ΔLEFS (*r* = −0.337, *P* = 0.039), and SANE (*r* = −0.382, *P* = 0.016) at the 24-month follow-up with lower ΔPROMs reported by those with older tears (Fig. 6).

**Fig. 7:**
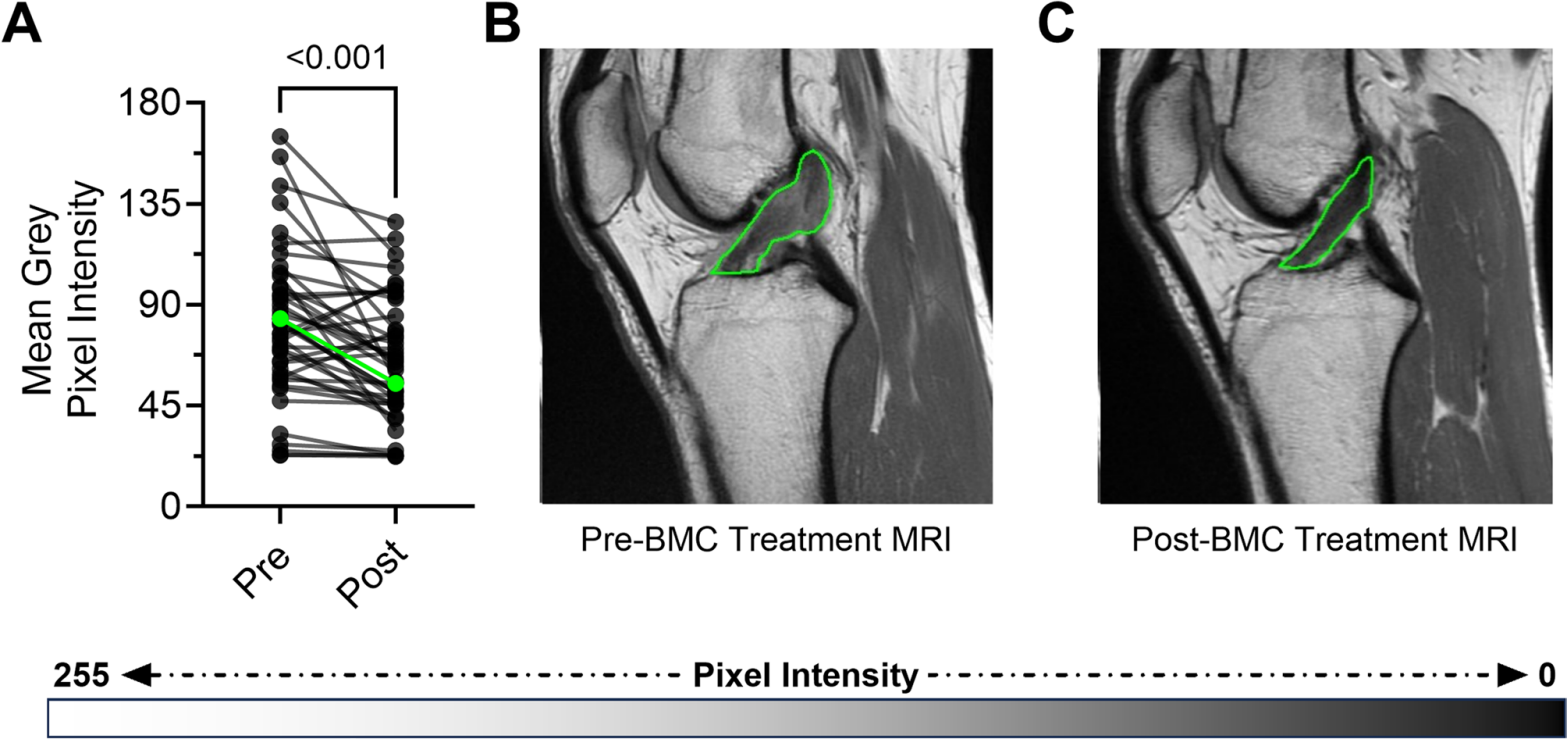
Change in ACL on MRI. Comparison of pixel intensity within ACL regions of interest pre- and post-BMC treatment **A**. Representative paired MRIs pre- (**B**) and post-BMC treatment (**C**). Paired t-test, *n* = 43

A significant pairwise decrease in pixel intensity, or MRI signal, was observed within the ACL regions-of-interest between the pre- and post-BMC treatment MRIs (*P* < 0.001) (Fig. 7). Interestingly, Telos measurements did not differ significantly (*P* > 0.05) from pre- to post-BMC but provided laxity measurements utilized at the time of evaluation. This is consistent with surgical ACL reconstruction studies that have shown no improvement in knee translation testing [29, 30].

## Discussion

The results of this randomized controlled trial highlight the potential of autologous BMC treatment for partial and full thickness non-retracted ACL tears. In this study, patients in the exercise group demonstrated significantly less pronounced improvements compared to those in BMC group. Improvements in LEFS and SANE scores were significantly higher in the BMC group, indicating that orthobiologics may offer superior functional recovery and patient satisfaction compared to exercise alone at the 3-month follow-up. This reflects past findings, that while exercise therapy can be effective for some patients, it often falls short in providing complete resolution of symptoms and restoring pre-injury levels of activity, especially in patients like those treated here who have sub-acute to chronic disability and are unable to return to play [31]. Filbay et al. [32, 33] published several papers describing a percentage of patients under conservative management showed ACL healing on MRI at both early and long-term follow-ups but had undergone early rehabilitation (within 4 weeks), whereas our patient population were 3 + months post ACL injury at a minimum which may provide rationale for our differing results.

Patients receiving BMC treatment reported better functional outcomes (LEFS) and higher levels of patient satisfaction (SANE) that were sustained over two years. Functional and pain metrics of IKDC and NPS improved significantly over baseline by the 3- and 6-month follow-ups, respectively, and were sustained through the remainder of the study period. A plausible explanation for IKDC and NPS outcomes between the two groups may be that the three-month cross-over was too soon to detect significant differences. Moreover, NPS was relatively low for both groups at baseline, making it difficult to detect differences between groups. The data also demonstrated that early intervention with BMC may be important, as there was a significant negative correlation between the age of ACL injury (AOI) and improvements in PROMs. This finding aligns with existing literature, which suggests that earlier treatment leads to better functional outcomes. Saraf et al. found that delays in treatment of ACL injuries for more than one year can result in poorer outcomes due to prolonged joint instability and ongoing damage [34]. These findings suggest that timely administration of orthobiologic treatment can lead to better outcomes, emphasizing the importance of early diagnosis and treatment of ACL injuries.

MRI findings following treatment with BMC demonstrated significant improvement which could indicate ACL integrity and fiber alignment. Image analysis revealed a marked decrease in the mean grey pixel intensity within the ACL regions of interest, indicating a reduction in injury-related hyperintensity. A decrease in signal intensity is consistent with the healing response, where the restoration of normal tissue architecture leads to a return toward baseline MRI appearance [33]. While interesting that Telos laxity measures did not show significant differences, a study by Lefevre et al. found that Telos was inferior to GNRB arthrometer as a diagnostic tool for partial ACL tears [35] and Sorensen et al. concluded that Telos was not recommended as a laxity measurement after treatment [30], both of which highlight the limitations of using this measure in our study.

The administration of PRP booster injections given to some patients experiencing a plateau in their healing may have added an additional variable that could independently cause an improved functional outcome over those not receiving them. However, the data reveals that those who received PRP booster injections reported less favorable outcomes overall, which is what prompted their administration in the first place. Specifically, patients who received PRP boosters reported significantly lower SANE scores at the later follow-ups compared to those who did not receive the boosters. Additionally, IKDC and LEFS scores trended lower amongst those receiving boosters, however still met the MCID threshold at all follow-ups. Possible explanations include that average AOI was longer in the booster group compared to those with no boosters and could be confounded by indication. Overall, while BMC treatment shows promise in improving functional recovery and patient satisfaction, the potential effects of PRP booster injections warrant further investigation.

In this study, the rate of ACL re-tears (showed evidence of healing on MRI prior to re-tear) among patients who received BMC treatment was approximately 4.16% (2 out of 48). This is comparable to the re-tear rate observed with traditional ACLR, which reports a 4.4% rate of graft rupture in the ipsilateral knee and 2.0% rate of ACL tear in the contralateral knee over a two-year period [36]. When excluding ACL re-injury, the overall non-ACL knee re-injury rate in our study was 6.25% (3 out of 48 patients). In contrast, traditional ACLR not only presents risks of graft rupture but also necessitates secondary surgeries in 7.3% of cases within the first two years due to complications or adverse events unrelated to graft re-injury such as meniscal tears (3.6%), intra-articular scarring (2.7%), chondral pathology (0.6%), and wound dehiscence (0.3%) [37]. We continue to follow this treated group long-term to determine longitudinal BMC retear rates and compare those against ACLR retear rates of five, ten, and fifteen years which have been reported at 12%, 27%, and 31%, respectively [36, 38, 39].

It is also important to consider the context of the four patients who chose to undergo surgical repair despite not having experienced re-injury. These patients did not suffer from ACL re-tears but rather opted for surgery possibly due to persistent symptoms, dissatisfaction with the initial outcomes, or personal preference for a more definitive solution. It indicates that while orthobiologic treatment may offer benefits in terms of reducing the risk of re-tear, it may not fully meet the needs of all patients, leading some to choose surgery and these should be considered BMC treatment failures with a rate of four in forty-eight or 8%. This is similar to data Inclan and Brophy reported in that 3%−22% of ACLRs lead to a graft failure from not only rupture, but also malposition and attenuation that can lead to repeated symptoms of instability and laxity [40].

ACLR surgery is the prevailing standard of care for complete ACL tears, aiming to restore knee stability by replacing the torn ligament with a graft. However, the results of this study offer promising insights into the potential of BMC treatment as a minimally invasive option for a distinct subset of ACL tears in patients looking to avoid surgery. Notably, the majority of patients who underwent BMC treatment reported being able to engage in strenuous or very strenuous activities regularly reaching 80% of participants at two years. This compares to surgical return to sport rates of 81% in a metanalysis [8], with MRI playing a potential role in predicting this in the future [41]. In this study no serious adverse events (SAE) were reported, and while the risks of SAE with ACL surgery are low (0.65%), infections and pulmonary embolism can occur [42]. BMC treatment preserves the native ACL structure, potentially maintaining the natural biomechanics and proprioception of the knee, which is speculated to be altered in surgical reconstruction. This minimally invasive technique can allow for rapid progression of rehabilitation. An advantage of BMC/MSC ACL treatment includes preventing the development of OA [16], since mild OA post-ACLR is common, with up to 33% developing moderate to severe OA compared to contralateral healthy knees [43].

Despite the promising results, the study has several limitations. While the study was appropriately powered, the sample size, lack of a placebo control group, and relatively short (3-months) crossover time point limits the ability to attribute the observed effects solely to BMC treatment. Allowing PRP boosters or repeat BMC treatments within the study group is a limitation that impacted a clear interpretation of the results, although were analyzed post-hoc to highlight their influence on the results. Additionally, the heterogeneous patient population and age of injury potentially influencing outcomes, however, may make findings more generalizable. Future research should aim to address these limitations by conducting larger, multicenter trials with homogeneous inclusion criteria. Moreover, mechanistic studies to elucidate the biological processes underpinning the clinical improvements observed with BMC treatment could provide deeper insights into its therapeutic potential.

## Conclusion

This randomized controlled, open-label, crossover trial demonstrates that autologous BMC combined with platelet products may be an effective treatment for partial thickness and full thickness non-retracted ACL tears. Patients receiving BMC treatment reported significant improvements in functional outcomes and pain relief compared to those undergoing exercise therapy, with benefits sustained over a 24-month period. This study provides a strong foundation for the continued investigation and clinical application of regenerative therapies in the management of ACL injuries.

## Supplementary Information

Supplementary Material 1.

Supplementary Material 2.

Supplementary Material 3. PROMs from the exercise therapy group and before and after crossing over to BMC treatment. Median values and interquartile ranges for IKDC (**A**), LEFS (**B**), NPS (**C**), and SANE (**D**) are shown. PROMs were compared over time using multiple Wilcoxon matched-pairs signed rank tests with Holm-Sidak correction. Post-BMC treatment follow-ups sharing a letter are statistically indistinguishable, whereas those with different letters are significantly different (*P* < 0.05).

Supplementary Material 4. ΔPROMs from recipients and non-recipients of a follow-up booster injection(s) for IKDC (**A**), LEFS (**B**), NPS (**C**), and SANE (**D**) at the 6-, 12- and 24-month follow-up are shown. Lines represent median values.

## Data Availability

Data used in this analysis during the current study may be made available upon reasonable request from the corresponding author.

## Abbreviations

ACL: Anterior cruciate ligament
ACLR: ACL reconstruction
AE: Adverse event
AM: Anteromedial
AOI: Age of injury
BMA: Bone marrow aspiration
BMC: Bone marrow concentrate
BMI: Body mass index
IKDC: International Knee Documentation Committee subjective knee form
IQR: Interquartile range
LCL: Lateral cruciate ligament
LEFS: Lower Extremity Function Survey
MCID: Minimal clinically important difference
MCL: Medial collateral ligament
MRI: Magnetic resonance imaging
MSC: Mesenchymal stem cell
NPS: Numeric pain scale
OA: Osteoarthritis
PD: Proton density
PL: Platelet lysate
PL: Posterolateral
PROM: Patient reported outcome measure
PRP: Platelet rich plasma
ROI: Region of interest
SAE: Serious adverse event
SANE: Single assessment numeric evaluation
SD: Standard deviation
