# Supplementary material for: Non-surgical treatment of anterior cruciate ligament tears with percutaneous bone marrow concentrate and platelet products versus exercise therapy: a randomized-controlled, crossover trial with 2-year follow-up
Source: BMC Musculoskelet Disord. 2025 Sep 30;26:882. doi: 10.1186/s12891-025-09153-2 (PMC12486544; doi:10.1186/s12891-025-09153-2)
Supplement: Supplementary file 1 — Supplementary Material 1. [file 12891_2025_9153_MOESM1_ESM.pdf]

Home Exercise Therapy Instructions

Weeks 1-6

Supine Runner's Stretch at Wall
reps: 10 sets: 3 hold: 5 Weekly: 3x Daily: 1x

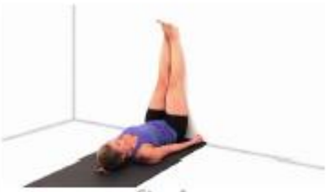

Step 1

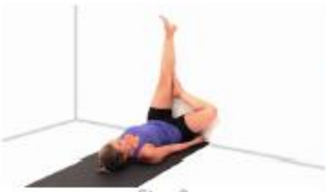

Step 2

Setup
- Begin lying on your back with your legs flat against wall.

Movement
- Slowly bend one leg, letting your knee to fall out to the side, and place the bottom of your foot on the inside of your leg.

Tip
- Make sure to keep your back flat against the ground and do not stretch through pain.

Modified Sidelying Quadriceps Stretch
reps: 10 sets: 3 hold: 5 Weekly: 3x Daily: 1x

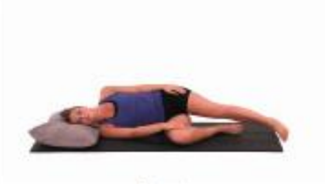

Step 1

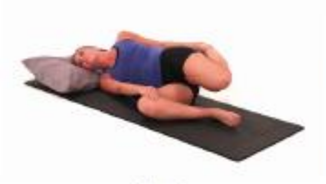

Step 2

Setup
- Being lying on your side with your knees bent at a 90 degree angle, holding your bottom knee with your bottom arm.

Movement
- Bend your top knee, then grasp your foot and gently pull your leg toward yourself until you feel a stretch in the front of your thigh.

Tip
- Make sure keep your shoulders facing forward and do not to let your trunk rotate during the exercise.

Seated Medial Patellar Glide
reps: 10 sets: 3 hold: 5 Weekly: 3x Daily: 1x

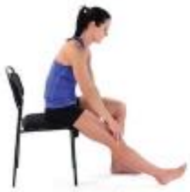

Step 1

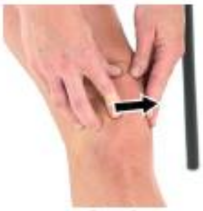

Step 2

Setup
- Sit on the edge of a chair with one leg stretched straight in front of your body and your heel resting on the ground.

Movement
- Place your fingers around your kneecap and lightly move it inward, then bring it back in place and repeat.

Tip
- Make sure to keep your leg muscles relaxed.

Seated Superior Patellar Glide
reps: 10 sets: 3 hold: 5 Weekly: 3x Daily: 1x

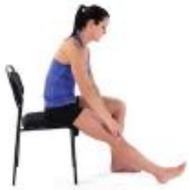

Step 1

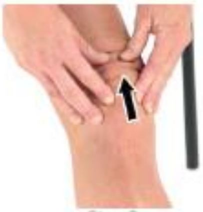

Step 2

Setup
- Sit on the edge of a chair with one leg stretched straight in front of your body and your heel resting on the ground.

Movement
- Place your fingers around your kneecap and lightly move it upward, then bring it back in place and repeat.

Tip
- Make sure to keep your leg muscles relaxed.

Seated Inferior Patellar Glide
reps: 10 sets: 3 hold: 5 Weekly: 3x Daily: 1x

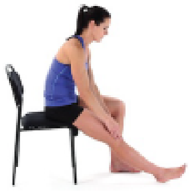
Step 1

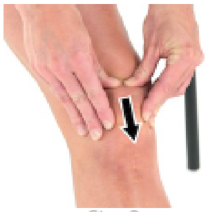
Step 2

Setup
- Sit on the edge of a chair with one leg stretched straight in front of your body and your heel resting on the ground.

Movement
- Place your fingers around your kneecap and lightly move it downward, then bring it back in place and repeat.

Tip
- Make sure to keep your leg muscles relaxed.

Supine Knee Extension Strengthening
reps: 10 sets: 3 hold: 5 Weekly: 3x Daily: 1x

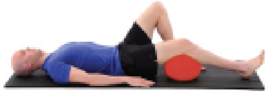
Step 1

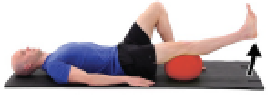
Step 2

Setup
- Begin lying on your back with one knee bent and the other resting on a ball.

Movement
- Straighten your knee by contracting your thigh muscles, keeping the back of your knee on the ball.

Tip
- Make sure not to arch your back during the exercise.

Prone Hamstring Curl with Anchored Resistance
reps: 10 sets: 3 hold: 5 Weekly: 3x Daily: 1x

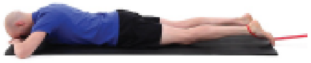
Step 1

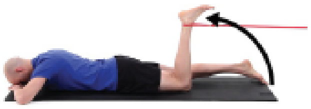
Step 2

Setup
- Begin lying face down with a resistance band secured around one ankle, facing away from the anchor point.

Movement
- Bend your knee, pulling your leg against the resistance toward your body.

Tip
- Make sure to keep your upper leg flat on the ground and do not arch your low back as you bend your knee.

Wall Squat with Resistance Loop
reps: 10 sets: 3 hold: 5 Weekly: 3x Daily: 1x

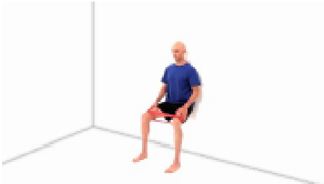
Step 1

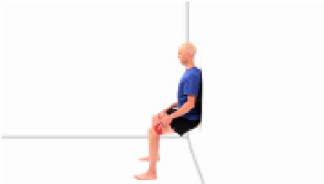
Step 2

Setup
- Begin in a standing upright position in front of a wall with your feet slightly wider than shoulder width apart and a resistance band around your knees.

Movement
- Lean back into a squat against the wall with your knees bent to 90 degrees and hold this position.

Tip
- Make sure your knees are not bent forward past your toes and keep your back flat against the wall. Maintain tension in the band throughout the exercise.

Wall Squat Hold with Ball
reps: 10 sets: 3 hold: 5 Weekly: 3x Daily: 1x

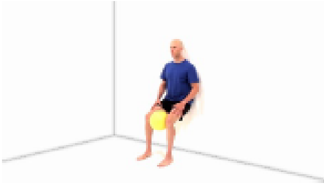
Step 1

Setup
- Begin in a standing upright position in front of a wall with your feet slightly wider than shoulder width apart and a ball in your hand.

Movement
- Lean back into a squat against the wall, placing the ball between your knees, and hold this position without letting the ball drop to the ground.

Tip
- Make sure your knees are not bent forward past your toes and keep your back flat against the wall during the exercise.

Alternating Single Leg Balance - Foot Behind
reps: 10 sets: 3 hold: 5 Weekly: 3x Daily: 1x

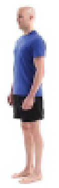
Step 1

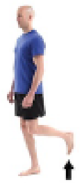
Step 2

Setup
- Begin in a standing upright position with your arms resting at your sides.

Movement
- Lift one foot off the ground behind your body, transferring your weight to the other leg. When you are balanced, slowly lower your foot to the floor and repeat on the other side.

Tip
- Make sure to maintain your balance and keep your back straight during the exercise.

Gastroc Stretch on Wall
reps: 10 sets: 3 hold: 5 Weekly: 3x Daily: 1x

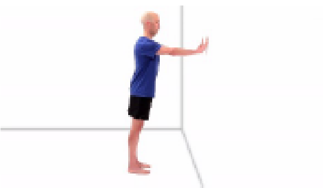
Step 1

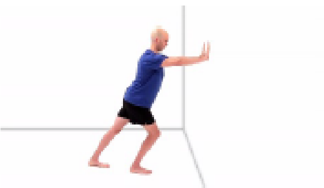
Step 2

Setup
- Begin in a standing upright position in front of a wall.

Movement
- Place your hands on the wall and extend one leg straight backward, bending your front leg, until you feel a stretch in the calf of your back leg and hold.

Tip
- Make sure to keep your heels on the ground and back knee straight during the stretch.

Soleus Stretch on Wall
reps: 10 sets: 3 hold: 5 Weekly: 3x Daily: 1x

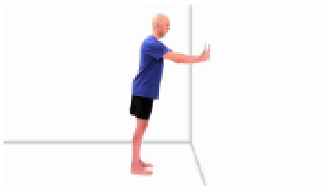
Step 1

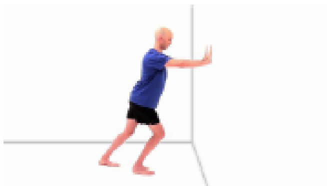
Step 2

Setup
- Begin in a standing upright position in front of a wall.

Movement
- Place your hands on the wall and extend one leg backward with your knee bent. Lean forward into the wall, until you feel a stretch in your lower calf and hold.

Tip
- Make sure to keep your heels on the ground and back knee bent during the stretch.

Set #2 Weeks 7-12+

Hip Adductors and Hamstring Stretch with Strap
reps: 10 sets: 3 hold: 5 Weekly: 3x Daily: 1x

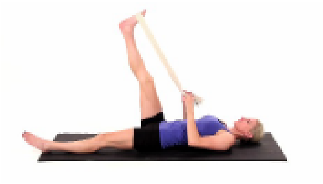
Step 1

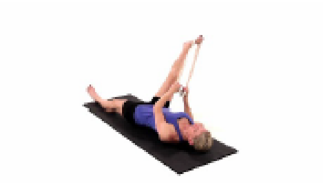
Step 2

Setup
- Begin by lying on your back with one leg straight, holding both ends of a strap secured around your other foot.

Movement
- Use the strap to pull your leg up toward your body, then straighten your leg and let it slowly lower out to the side. You should feel a stretch in the inside of your thigh.

Tip
- Make sure to keep your low back flat against the floor.

Standing Quad Stretch with Strap
reps: 10 sets: 3 hold: 5 Weekly: 3x Daily: 1x

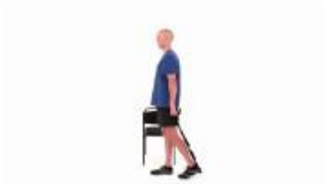
Step 1

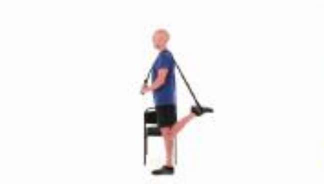
Step 2

Setup
- Begin in a standing upright position holding on to a stable object at your side with a strap looped around one foot, holding the end in your hand.

Movement
- Pull the strap over your shoulder and pull your leg upward until you feel a stretch in the front of your thigh and hold.

Tip
- Make sure to keep your back straight and maintain your balance during the stretch.

Standing Hamstring Curl with Resistance
reps: 10 sets: 3 hold: 5 Weekly: 3x Daily: 1x

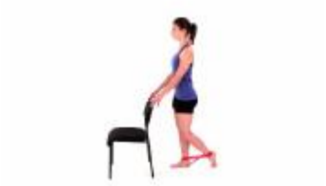
Step 1

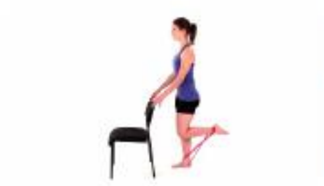
Step 2

Setup
- Begin in a standing upright position with a resistance band looped under the bottom of one foot and around your opposite ankle, holding onto a chair for support.

Movement
- Bend your knee, pulling your foot upward against the resistance, then slowly lower it back down and repeat.

Tip
- Make sure to keep your back straight and maintain your balance during the exercise.

Deadlift with Resistance
reps: 10 sets: 3 hold: 5 Weekly: 3x Daily: 1x

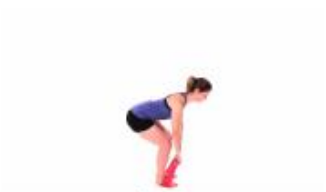
Step 1

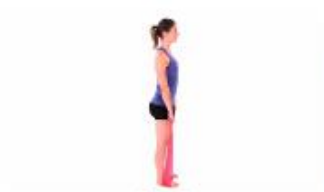
Step 2

Setup
- Begin in a standing upright position with holding both ends of a resistance band that is anchored under your feet.

Movement
- Bend at your hips and knees, lowering your arms toward the ground, then stand back up, engaging your back and thigh muscles and pulling up on the resistance band with your arms straight.

Tip
- Make sure to keep your abdominals tight and back straight during the exercise.

Standing Terminal Knee Extension with Resistance
reps: 10 sets: 3 hold: 5 Weekly: 3x Daily: 1x

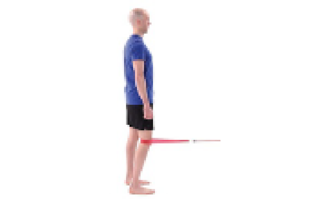
Step 1

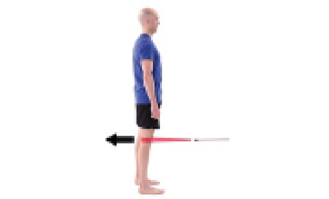
Step 2

Setup
- Begin in a standing upright position with one knee slightly bent and a resistance band secured just above it. You should be facing the anchor point.

Movement
- Gently straighten your knee, pulling against the resistance band.

Tip
- Make sure to keep your heel on the ground, your hips level, and do not overextend your knee.

Squat with Chair Touch
reps: 10 sets: 3 hold: 5 Weekly: 3x Daily: 1x

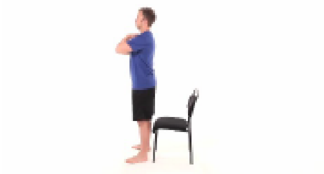
Step 1

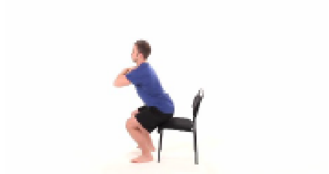
Step 2

Setup
- Begin in a standing upright position in front of a chair.

Movement
- Lower yourself into a squatting position so you lightly touch the chair, then repeat.

Tip
- Make sure to maintain your balance during the exercise and do not let your knees bend forward past your toes.

Step Up

reps: 10 sets: 3 hold: 5 Weekly: 3x Daily: 1x

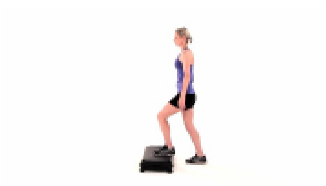
Step 1

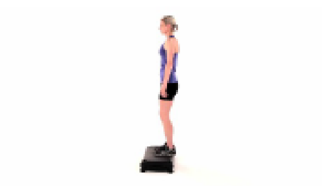
Step 2

Setup

- Begin standing with a small step or platform in front of you.

Movement

- Step up onto the platform with one foot then follow with your other foot. Return back down to the starting position and repeat.

Tip

- Make sure to maintain good posture during the exercise and do not let your knee bend forward past your toe as you step up.

Step Downs

reps: 10 sets: 3 hold: 5 Weekly: 3x Daily: 1x

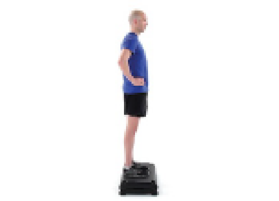
Step 1

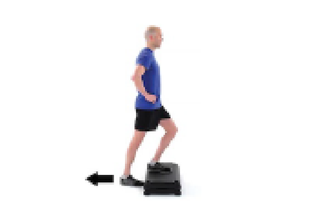
Step 2

Setup

- Begin in a standing position with one leg on a step and the other hanging backward off the edge.

Movement

- Bend your knee, lowering your foot toward the floor, then return back to the starting position by pushing upward on your front leg.

Tip

- Make sure to maintain your balance during the exercise and do not let your trunk lean forward.

Sidestepping with Resistance at Thighs

reps: 10 sets: 3 hold: 5 Weekly: 3x Daily: 1x

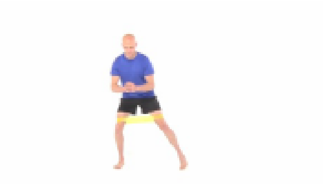
Step 1

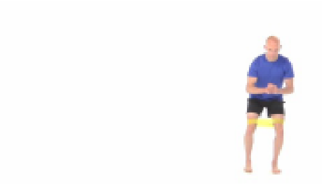
Step 2

Setup

- Begin in a standing position with a resistance loop secured around your knees. Bend your knees slightly so you are in a squatting position.

Movement

- Slowly step sideways, maintaining tension in the band.

Tip

- Make sure to keep your feet pointing straight forward and do not let your knees collapse inward during the exercise.

Seated Plantar Fascia Mobilization with Small Ball

reps: 10 sets: 3 hold: 5 Weekly: 3x Daily: 1x

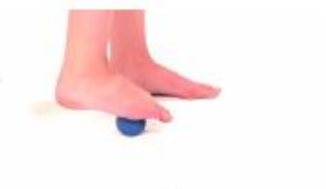
Step 1

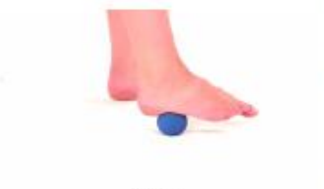
Step 2

Setup

- Begin sitting in a chair with your foot resting on a small ball.

Movement

- Gently roll the middle of your foot forward and backward over the ball, in between the ball of your foot and your heel.

Tip

- Make sure to use just enough pressure that you feel a stretch but no pain.

Calf Mobilization with Small Ball
reps: 10 sets: 3 hold: 5 Weekly: 3x Daily: 1x

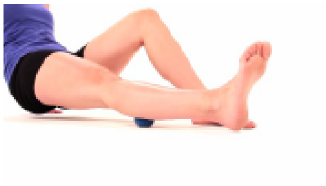

Step 1

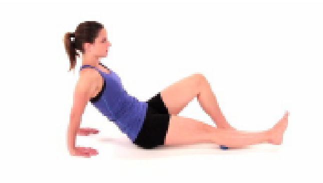

Step 2

Setup

- Begin sitting in an upright position on the floor with one knee bent and your other leg straight, resting with a tennis ball under your calf.

Movement

- Lift your body off the ground and slowly roll your calf over the tennis ball.

Tip

- Make sure to apply just enough pressure to feel a stretch but no pain.

ITB Stretch at Wall
reps: 10 sets: 3 hold: 5 Weekly: 3x Daily: 1x

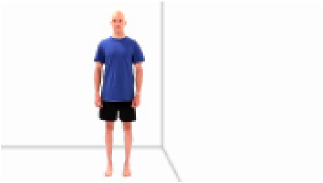

Step 1

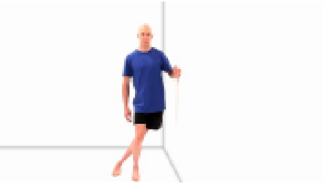

Step 2

Setup

- Begin in a standing upright position next to a wall. Cross your outside leg over the other and place your arm against the wall.

Movement

- Gently push your hip toward the wall until you feel a stretch in the side of your leg and hold.

Tip

- Make sure not to lean forward or backward.
