# Supplementary material for: Non-surgical treatment of anterior cruciate ligament tears with percutaneous bone marrow concentrate and platelet products versus exercise therapy: a randomized-controlled, crossover trial with 2-year follow-up
Source: BMC Musculoskelet Disord. 2025 Sep 30;26:882. doi: 10.1186/s12891-025-09153-2 (PMC12486544; doi:10.1186/s12891-025-09153-2)
Supplement: Supplementary file 2 — Supplementary Material 2. [file 12891_2025_9153_MOESM2_ESM.docx]

**Supplementary 2 –** Crossover to BMC treatment (exercise therapy group) PROMs.

| **Crossover to BMC Treatment** | | | | | | |
| --- | --- | --- | --- | --- | --- | --- |
| **PROM** | **Baseline (n = 14)** | **1-Month (n =13)** | **3-Month (n = 14)** | **6-Month (n = 14)** | **12-Month (n = 13)** | **24-Month (n = 14)** |
| IKDC | 62.4 [52.3 to 67.3]^A^ | 70.1 [65.0 to 73.6]^B^ | 82.8 [76.2 to 89.1]^C^ | 86.2 [83.3 to 89.4]^C^ | 88.5 [80.5 to 93.7]^CD^ | 94.3 [88.2 to 96.6]^D^ |
| LEFS | 56.5 [49.3 to 49.3]^A^ | 66.0 [59.0 to 69.0]^A^ | 75.0 [67.8 to 76.3]^B^ | 74.5 [71.0 to 77.3]^B^ | 77.0 [70.0 to 79.5]^BC^ | 77.5 [74.3 to 79.3]^C^ |
| NPS | 1.5 [0.8 to 2.5]^A^ | 0.0 [0.0 to 1.0]^AB^ | 0.0 [0.0 to 1.0]^AB^ | 0.0 [0.0 to 1.0]^AB^ | 0.0 [0.0 to 0.0]^AB^ | 0.0 [0.0 to 0.0]^B^ |
| SANE | - | 10.0 [0.0 to 30.0]^A^ | 60.0 [42.5 to 77.5]^B^ | 80.0 [70.0 to 90.0]^B^ | 90.0 [80.0 to 99.0]^BC^ | 90.0 [85.0 to 100.0]^C^ |
| Values presented as median [interquartile range], n = displayed). PROM-specific follow-up timepoints sharing a letter are indistinguishable via Wilcoxon matched-pairs signed rank test (adjusted P < 0.05), whereas values with different letters were significantly different (P<.05). | | | | | | |
