# Supplementary figures and images for: Non-surgical treatment of anterior cruciate ligament tears with percutaneous bone marrow concentrate and platelet products versus exercise therapy: a randomized-controlled, crossover trial with 2-year follow-up
Source: BMC Musculoskelet Disord. 2025 Sep 30;26:882. doi: 10.1186/s12891-025-09153-2 (PMC12486544; doi:10.1186/s12891-025-09153-2)

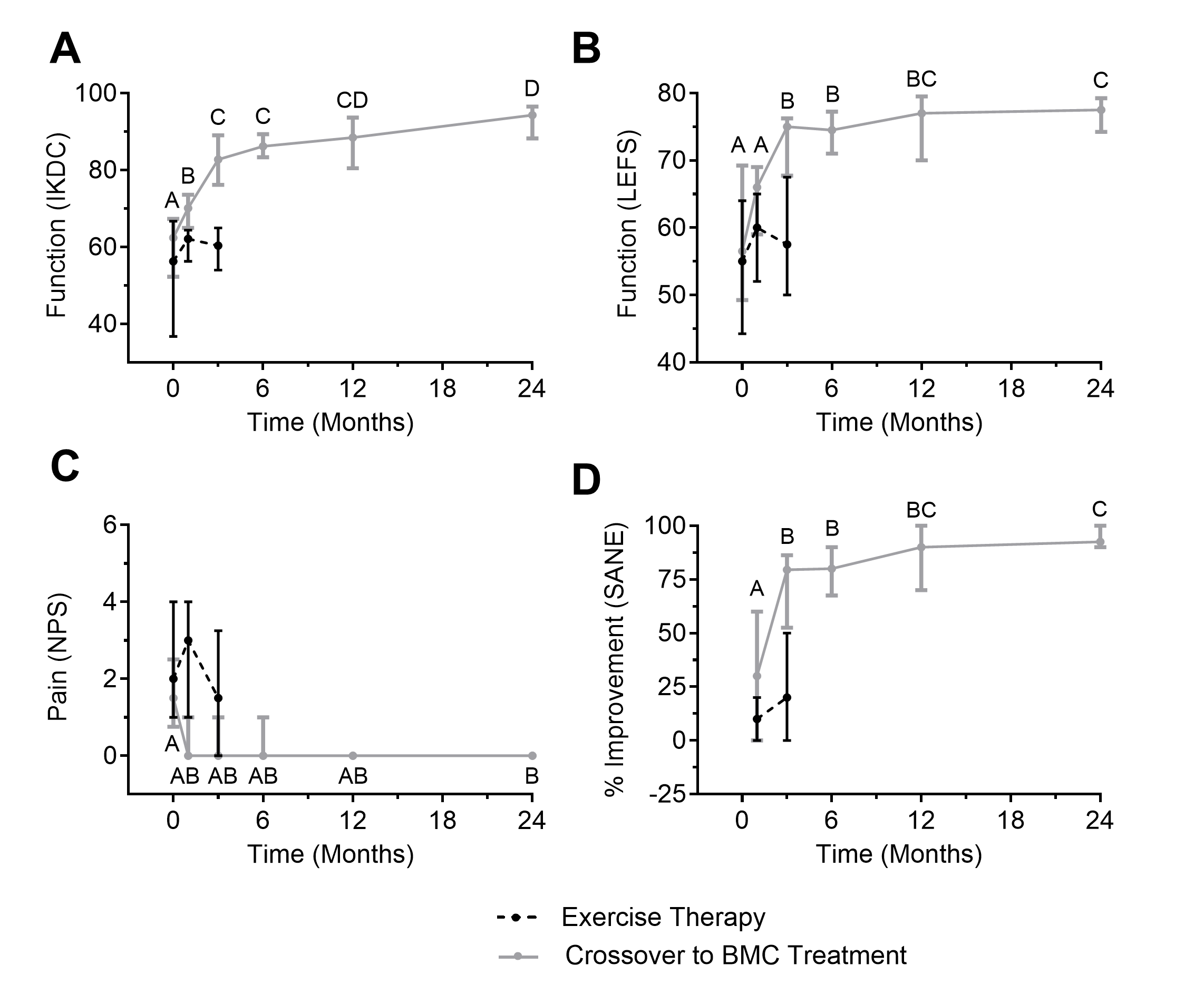

Supplement: Supplementary file 3 — Supplementary Material 3. PROMs from the exercise therapy group and before and after crossing over to BMC treatment. Median values and interquartile ranges for IKDC (A), LEFS (B), NPS (C), and SANE (D) are shown. PROMs were compared over time using multiple Wilcoxon matched-pairs signed rank tests with Holm-Sidak correction. Post-BMC treatment follow-ups sharing a letter are statistically indistinguishable, whereas those with different letters are significantly different (P < 0.05). [file 12891_2025_9153_MOESM3_ESM.tif]

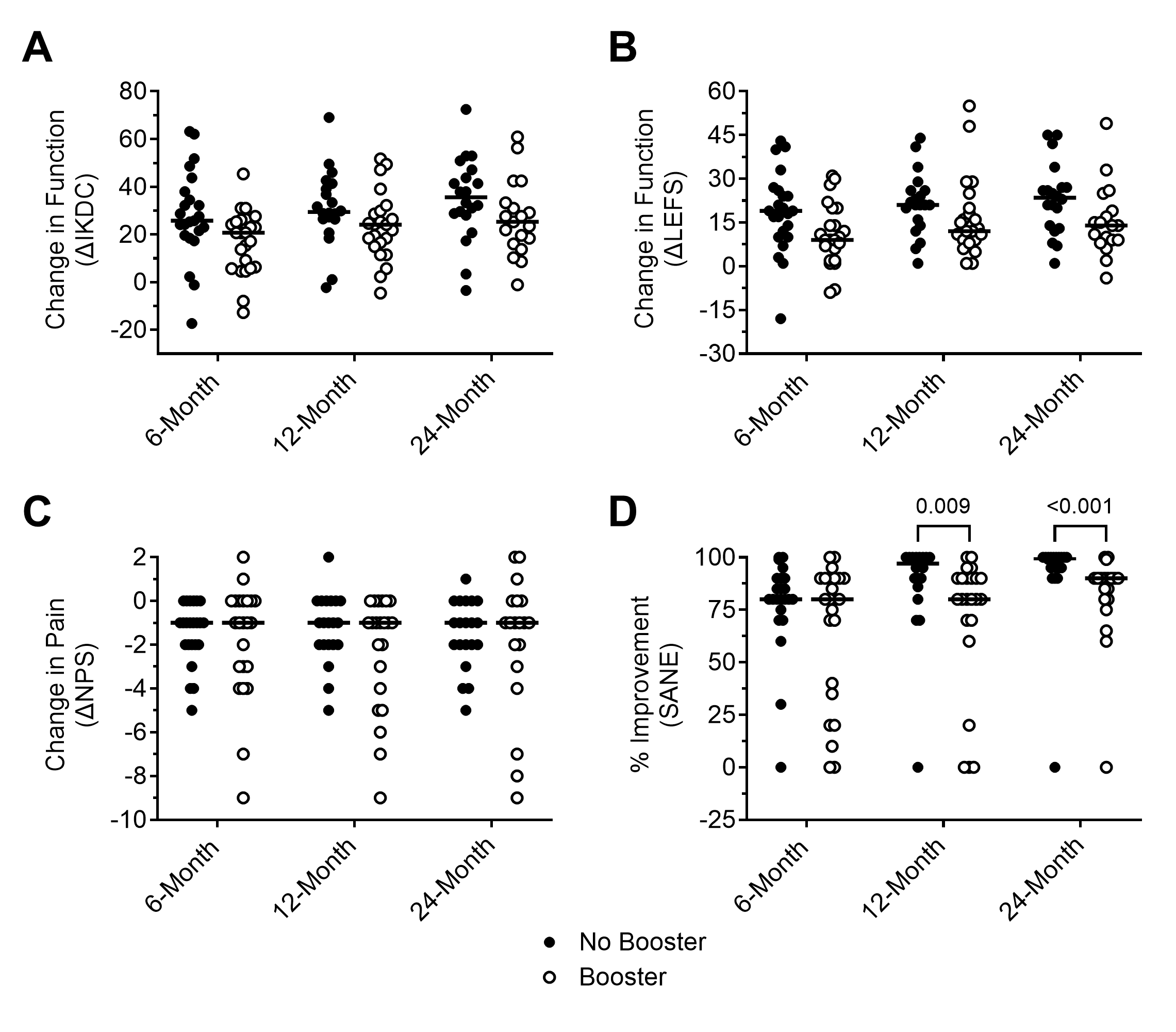

Supplement: Supplementary file 4 — Supplementary Material 4. ΔPROMs from recipients and non-recipients of a follow-up booster injection(s) for IKDC (A), LEFS (B), NPS (C), and SANE (D) at the 6-, 12- and 24-month follow-up are shown. Lines represent median values. [file 12891_2025_9153_MOESM4_ESM.tif]
